# Laughter and its role in the evolution of human social bonding

**DOI:** 10.1098/rstb.2021.0176

**Published:** 2022-11-07

**Authors:** R. I. M. Dunbar

**Affiliations:** Department of Experimental Psychology, University of Oxford, Radcliffe Observatory Quarter, Oxford OX2 6GG, UK

**Keywords:** Duchenne laughter, social grooming, group size, endorphins

## Abstract

In anthropoid primates, social grooming is the principal mechanism (mediated by the central nervous system endorphin system) that underpins social bonding. However, the time available for social grooming is limited, and this imposes an upper limit on the size of group that can be bonded in this way. I suggest that, when hominins needed to increase the size of their groups beyond the limit that could be bonded by grooming, they co-opted laughter (a modified version of the play vocalization found widely among the catarrhine primates) as a form of chorusing to fill the gap. I show, first, that human laughter both upregulates the brain's endorphin system and increases the sense of bonding between those who laugh together. I then use a reverse engineering approach to model group sizes and grooming time requirements for fossil hominin species to search for pinch points where a phase shift in bonding mechanisms might have occurred. The results suggest that the most likely time for the origin of human-like laughter is the appearance of the genus *Homo* *ca* 2.5 Ma.

This article is part of the theme issue ‘Cracking the laugh code: laughter through the lens of biology, psychology and neuroscience’.

## 1. Introduction

Although we share laughter with the great apes [1], and the vocalization itself is homologous with the play invitation pant of catarrhine primates [2,3], human laughter differs from that found in nonhuman primates in the fine detail of both its structure and its physiological characteristics [3–5]. Structurally, the difference is marked by a shift from an exhalation-inhalation sequence in monkeys and apes to an exhalation-only sequence with no intervening inhalation in humans. This has the effect of emptying the lungs and placing intense physiological strain on the diaphragm and chest wall muscles [6], such that in sustained bouts of the kind that occur in relaxed social laughter we can be left gasping for breath. This form of (Duchenne) laughter is involuntary and highly contagious (we are up to 30 times more likely to laugh when we watch a comedy video in a group than if we watch the same video alone [3,7]). Indeed, laughter can occur even in the absence of any obvious stimulus if someone else laughs (e.g. the ‘giggles’) [4], creating a distinctive form of group chorusing not observed in any other primates in an affiliative social context. Humans also have a wider variety of laughs than other primates (Duchenne and non-Duchenne laughs, each with several subtypes); these differ in structure, involve different brain regions [8] and can carry very different meanings (appeasement, politeness, happiness, interest, etc) [3,9]. Casual human conversations are littered with laughter, to the point where conversations that lack laughter quickly become hard work.

That laughter is such an integral component in our social interactions begs questions about its function and evolutionary origins. Most research on the functions of laughter has focused on the information being broadcast by the person laughing or its role in inducing positive affect in the listener, thereby facilitating interaction or reducing threat [10–18]. However, it has also been suggested that human laughter of the involuntary Duchenne type plays a central role in group bonding, at least on the intimate conversational scale [19–21]. As such, it seems like a plausible candidate to fill the gap between primate social grooming and other evolutionarily more recent social bonding behaviours such as singing, dancing, feasting and storytelling [7,19,20,22]. There are good grounds for supposing that laughter evolved before these other bonding behaviours: first, only laughter is shared with the great apes and, second, laughter has a strongly involuntary component to it whereas all these other behaviours are under explicitly voluntary control (and/or depend on language). This suggests that laughter has very deep evolutionary roots whereas the other bonding behaviours are of much more recent origin [20]. At least as far as music is concerned, we can identify on anatomical grounds a date (the appearance of archaic humans, *ca* 600 ka) as its likely time of origin [23], while, on strictly cognitive grounds, fully modern storytelling probably had to wait for the appearance of anatomically modern humans (some time after 250 ka) [24].

The hypothesis explored here is straight forward: at some point during the course of hominin evolution it (i) became necessary to add a further mechanism to the process of group bonding because the conventional primate mechanism of social grooming reached a limit set by the time available to devote to it, and (ii) that this additional mechanism was laughter because (iii) laughter triggers the same neurophysiological mechanism that underpins primate social bonding without (iv) being subject to the same time and intimacy constraints as grooming. To explore this, I first establish that grooming imposes constraints on primates' ability to bond large groups. I then show that, in modern humans, laughter triggers the same endorphin system that grooming does and, in doing so, boosts the sense of belonging (social bonding). Finally, I use a reverse engineering approach to examine how the social bonding demand is likely to have increased over the course of human evolution in order to identify likely time points at which there would have been a serious time constraint. I suggest that these can be reduced to just two critical time points (the appearance of early *Homo* around 2.4 Ma and the appearance of archaic humans around 600 ka), and offer some suggestions as to why the first is the more likely.

It is important to be clear that the issue here is not replacement of one bonding mechanism by another, but rather accretion or supplementation by a series of successive mechanisms each designed to solve a different pinch point [20]. The claim explored here is that laughter was a transitional phase between conventional primate bonding mechanisms and the more culture-based bonding behaviours found in modern humans. Laughter is not, however, a precursor for language since the anatomical and physiological mechanisms involved are very different to those involved in speech. That we now use language in the form of jokes to stimulate laughter is not relevant to the *function* of laughter or when it evolved. Jokes are simply one way we can control the elicitation of laughter, but not the only way.

## 2. Constraints on grooming

Like all catarrhine primates, humans live in stable social groups [25,26]. These require considerable investment in behavioural processes that create bonded relationships so as to maintain their stability and cohesion through time [27]. The principal mechanism for creating bonded relationships in primates is social grooming ([28,29]; see the electronic supplementary material for more detail), acting through the brain's endorphin system [30,31]. The sweeping hand actions used when leafing through the fur during grooming activate a set of low-threshold afferent c-fibre mechanoreceptors (CLTMs) that are widely distributed throughout the hairy skin [32–34]. These activate c-tactile (CT) neurons, a set of unmyelinated, low velocity, no-return-loop peripheral nerves that project to the posterior insular cortex (rather than to the somatosensory cortex that is the primary target of the myelinated touch and nociception nerves). From here, they trigger upregulation of the brain's endorphin system. The CTLM receptors are highly specialized and respond only to light slow stroking (soft touch) at a very specific speed (3 cm s^−1^) [35–37]. The opiate-like response produced by the activation of the endorphin system gives rise to a sense of mild euphoria, heightened analgesia, warmth, calmness, relaxation and trust, thereby inducing a sense of emotional closeness and bondedness between the individuals concerned [38–41].

Positron emission tomography (PET-scanning) has demonstrated that, in humans, light, slow stroking results in endorphin uptake throughout most of the brain (except the visual system) [42], indicating that the same system is still involved in human social touch. The importance of physical touch in facilitating a sense of bondedness in humans is evidenced by the correlation between emotional and social closeness and how much of the body surface it is permissible to touch in a variety of different cultural and ethnographic contexts [43,44].

**Figure 1.**
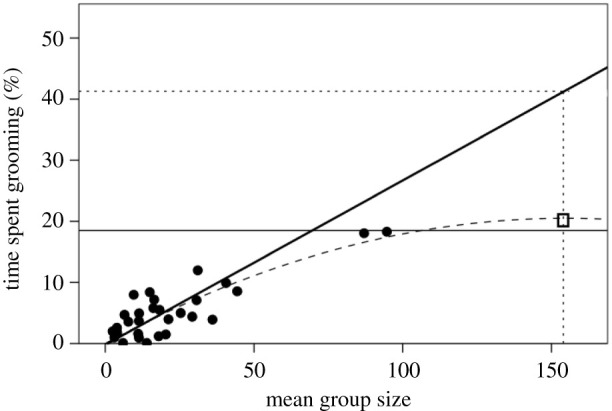
Mean per cent of daytime devoted to social grooming as a function of mean group size for individual anthropoid primate genera. The dashed line is the best fit quadratic regression (*r*^2^ = 0.684, *F*_2,26_ = 29.2, *p* < 0.0001); the heavy solid line is the reduced major axis linear regression for groups of less than 45 (ordinary least squares regression: *r*^2^ = 0.599, *F*_1,25_ = 14.0, *p* = 0.001). The thin solid line demarcates the upper limit on observed grooming time at 18.5% of total daytime. The dotted lines plot the predicted grooming time for humans (*Homo sapiens*) living in their observed mean group size of 154 [45]. The square identifies the mean time devoted to social interaction (predominantly conversation, of course) in seven widely different human societies [45]. The data are given in the electronic supplementary material table S1.

In nonhuman primates, the time devoted to social grooming increases from less than 1% of the day in the least social species that live in the smallest social groups (usually pair-living) to approximately 20% in the most social species that live in the largest groups [28] (figure 1). There are two points to note. First, there is a broadly linear increase in the time devoted to social grooming as group size increases; second, the time devoted to socializing reaches an asymptotic value at approximately 18.5% of the day (as indicated by the dashed nonlinear regression line). Although both linear and quadratic regressions give significant fits to the data (*p* < 0.001 in each case), the quadratic regression provides a rather better fit (*r*^2^ = 0.614 versus *r*^2^ = 0.713, respectively). The substantive issue, however, is that no primate species devotes more than 18.5% of its daily time budget to social grooming.

The asymptote at 18.5% is owing to the competing demands of other activities that animals must undertake to meet their daily nutritional and other requirements, including travel between feeding sites, time spent feeding (searching for, harvesting and processing food items) and time spent resting [46]. Time spent resting refers to time that animals are forced to spend resting (enforced resting time) owing to a combination of the demands of food processing (e.g. ‘rumination’ in folivores) and time when ambient temperatures rise to such a level that the heat load created by activity exceeds the animal's capacity to dissipate heat [47]. Animals cannot compromise on these components of their time budgets since they directly impact on their ability to survive. Time available for social interaction—and hence bonding both dyadic relationships (alliances) and social groups—is whatever is left over in the 12 hour waking day characteristic of all diurnal primates. This sets an upper limit on the size of group that can be bonded [46]. (Note that all other activities, including territory defence, evicting intruders, territorial advertising, scent marking, defecating etc, take up a negligible amount of time, and can be ignored for these purposes.) That primates, in particular, face serious time constraints is attested to by observational data on maternal time budgets (mothers are obliged to increase feeding time and reduce social time as the infant's lactational demand increases with age [48,49]) and by field experiments that have manipulated food availability (with the reduction in feeding time as a result of artificial feeding being converted into social grooming) [50].

Figure 1 suggests that the asymptotic effect only begins to impose a serious limit on animals' time budgets once group size becomes large. For an asymptotic regression, the point at which further increases in the independent variable produce declining improvements in the dependent variable is the point on the *y*-axis that is 1/e down from the asymptote [51]. In figure 1, this corresponds to a group size of 44.3 (the exact mean group size for both *Papio* and *Pan*, the two most intensely social primate genera [52]). If we exclude group sizes greater than 45, there is a significant linear regression for the remaining data on the left-hand side of the graph (*r*^2^ = 0.434, *p* = 0.001). However, since the data are better described as bivariate uniform rather than bivariate normal (as required for conventional ordinary least squares regression), a reduced major axis (RMA) regression will provide a better fit (see the electronic supplementary material). The RMA regression for these data is:

2.1 %groom=−0.223+0.269×group size.

It is notable that the two taxa which lie on the extreme right-hand side of the graph (*Papio hamadryas* and *Theropithecus gelada*) both have a form of social structure (small, stable, semi-independent reproductive units or harems, nested within large unstable bands) that is unique among the primates [26,53–55]. It seems that they evolved this system in response to the stresses created by having to live in very large social groups [56], a problem they solve by substructuring the group so as to create a multilevel social system that exploits the capacity to form temporary herds and at the same time allows the animals to defuse the stresses of group-living by dispersing when it is safe to do so [26,57].

Equation (2.1) predicts that humans living in the mean observed group size for contemporary humans of 154 [45] would need to devote 41.2% of their waking day to grooming time if they were to bond their groups by social grooming alone (the horizontal dotted line in figure 1). In fact, a sample of time budgets from seven societies drawn from a wide range of cultures gives an average of 20% of the day devoted to social interaction [58]. In other words, it seems that modern humans use the same amount of time as the most social nonhuman primates, but somehow manage to use it more efficiently in order to ‘groom’ with more individuals.

Grooming is extremely costly in terms of the time that has to be invested in a relationship to maintain it at a specific degree of emotional closeness, and this is as true for humans as it is for other primates [59–61]. Given that a minimum time investment is needed in each relationship to ensure that it is socially functional and that the time available for social interaction is limited, there will inevitably be an upper limit on the size of social group that can be bonded by this means. This limit seems to occur at approximately 50 individuals, roughly the upper limit on the *mean* size of social groups in nonhuman primates [26]. Note that this does *not* mean that individual groups cannot be larger: primate [46,62,63], and human [64], groups are dynamic nonlinear oscillators designed to target a particular mean size dictated by the trade-off between fertility and the demands of the local habitat [57,62,63]. Although each species has a target size suited to the habitats it is adapted to, its groups cannot undergo fission the moment they exceed the optimal size: they have to wait until their size is at least twice the minimum set by the habitat's riskiness [46]. As a result, groups oscillate within a range either side of the optimum value, and thus unavoidably incur significant costs in terms of both fertility [56,57,62,63] and group stability [46,65] during the time they spend at either end of the oscillator.

So long as the ideal group size is less than approximately 50 animals, grooming demand can be met fairly easily [28,29,46]. However, as later hominins evolved away from their australopithecine ancestors, their occupation of increasingly terrestrial (and hence more predator risky) habitats inevitably demanded a progressive increase in group size beyond the levels that could be bonded by grooming [20]. Some additional mechanism was, therefore, needed that by-passed the constraints imposed by the intimacy of social grooming so as to allow the size of the bonded group to continue increasing [20,66]. The constraint imposed by grooming arises from its physical intimacy: in essence, it is a one-on-one activity that it is normally uni-directional (A grooms B, with roles being reversed from time to time in reciprocated grooming sessions). Consequently, the only way the glass ceiling can be breached is by finding behaviours that trigger the endorphin system without need of direct physical contact so as to be able to ‘groom’ with several individuals simultaneously.

**Figure 2.**
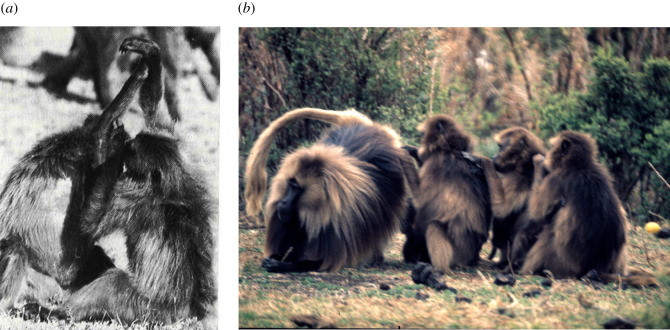
(*a*) Crossed-arms mutual grooming in gelada baboons: two animals lock wrists together and groom each other's upper arm or armpit. This form of distinctive of mutual grooming is common in gelada and has been reported in chimpanzees [67]. I have not observed it in the field in *Papio*, *Colobus* or *Chlorocebus* monkeys. (*b*) A grooming chain involving a gelada harem male and his three females. Photos, Robin Dunbar.

**Figure 3.**
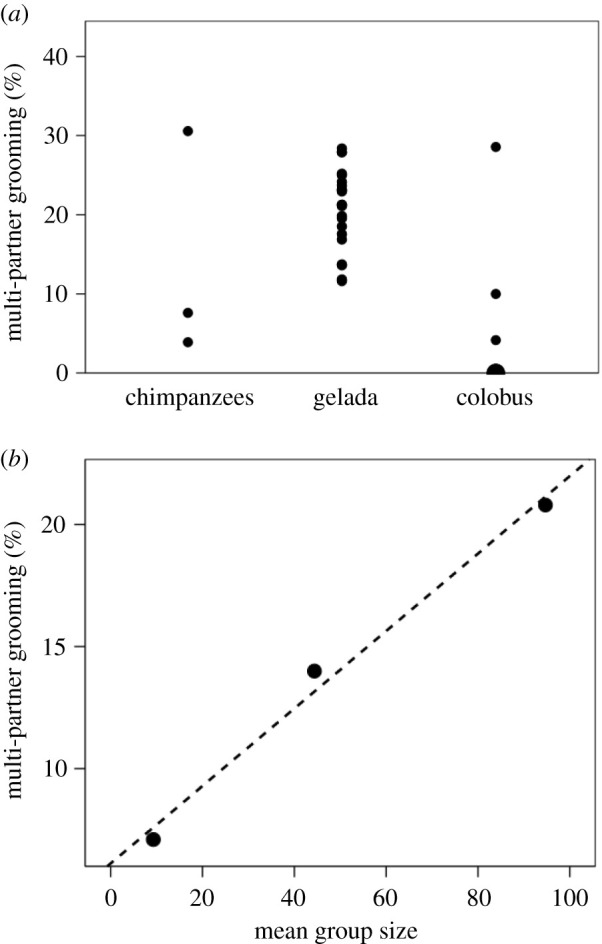
(*a*) Mean frequency of multi-partner grooming sets in individual groups of three catarrhine primates. Multi-partner grooming includes mutual grooming (figure 2, left photo), grooming chains (figure 2, right photo) and more complex forms. The large symbol in the bottom right corner indicates that two datapoints are superimposed. All data are from wild populations. The chimpanzee data represent both common chimpanzee and bonobo. The data are given in the electronic supplementary material, table S2. (*b*) Mean frequency of multi-party grooming for each species in (*a*), plotted against mean social group size (group for colobus, community for chimpanzees and band for gelada, respectively). Group size data from the electronic supplementary material, table S1.

Mutual grooming (in which two animals groom each other simultaneously: figure 2, left) may be a potential solution since both partners get an endorphin hit simultaneously rather than just the recipient, thereby making more effective use of the available time. Chain grooming (figure 2, right) may likewise increase the efficiency of social time use for the same reason, although each individual still, in effect, only grooms with (i.e. grooms and is groomed by) one individual no matter how long the chain, so that the effective benefit may be the same as that for mutual grooming. However, mutual grooming is relatively rare, and more complex multi-individual grooming formations rarer still. Figure 3*a* plots the proportion of all grooming time during which pairs of individuals mutually groomed or formed multi-individual grooming sets in three genera of primates. The majority of these cases involve mutual grooming or three-way formations in which two animals simultaneously groomed a third (electronic supplementary material, table S2). More complex formations involving more individuals are extremely rare, perhaps suggesting that knowing *who* is grooming you is critical. In other words, grooming is a form of bilateral focussed attention (as it clearly is in humans, where being caressed by several individuals simultaneously is generally considered less pleasant than being caressed by one in whom one has a special interest).

In this sample of primates, multiple-partner grooming accounts for less than 20% of all grooming time, and in most cases less than 10%. More importantly, it seems that multiple-partner grooming is a linear function of species typical social group size (figure 3*b*), suggesting that this may well represent an attempt to solve the increasing demand for grooming time as the size of the social group, and hence the stresses of group-living [56], increase but the available time budget remains fixed. Mutual grooming and grooming chains effectively double the use of grooming time, in effect allowing two individuals to be groomed for the price of one. If simple exposure to being groomed is the issue, then, for a genus like *Pan* that averages 8.4% of its day grooming and devotes 14% of that to forms of simultaneous mutual grooming, effective grooming time would be 8.44 × 1.140 = 9.6% (placing this genus very close to the RMA regression line in figure 1), while it would increase gelada grooming time from 18.3% to 22.0% (significantly closer to the 25.3% that the RMA regression would predict for this taxon). In the *Colobus* case, however, a 7% uplift owing to mutual grooming would have only a marginal effect, increasing grooming time from 4.9% to a very modest 5.3%.

While multi-partner grooming may help to increase the number of individuals who receive the appropriate levels of endorphin activation in some highly social primates, these uplifts would be insufficient to allow humans to bond significantly larger groups than those already found among primates unless greater than 30% of human *grooming* interactions were multi-partner. There is no evidence that this is ever the case in humans. When active soft touch occurs in humans, it is almost always one-to-one and considered an extremely intimate behaviour, as it is in monkeys and apes. Something that works more effectively with larger numbers of individuals while avoiding the constraints imposed by the intimacy of active physical touch would seem to be required. (Extra time spent in passive physical contact would not be sufficient for this, as it does not activate the CT neuron system. In any case, adult humans do not spend significant quantities of time in physical contact with anyone other than a few intimate partners [43,44,68].) I suggest that laughter as a form of chorusing was the solution that made it possible to break through the grooming constraint.

## 3. Laughter, endorphins and bonding

Grooming works as a bonding mechanism because it upregulates endorphins in the brain. Endorphins have been shown to be explicitly involved in grooming in monkeys [30,31], and to be involved in mother–infant bonding [69]. Endorphins are a family of opioid neuropeptides that act as neurotransmitters. Of the three main types (endorphins, enkephalins and dynorphins), β-endorphins are of particular interest because they seem to be uniquely involved both in the modulation of pain and the facilitation of social bonding. They are produced in the hypothalamic nuclei and the mesolimbic structures involved in reward [70,71], and have a particular affinity for the μ (and to a lesser extent κ) opioid receptors that are found throughout the brain (other than the visual system in the occipital lobe) [4]. Because endorphins do not cross the blood–brain barrier [72], assaying central endorphin upregulation requires either lumber puncture (to measure endorphin output in cerebrospinal fluid) or PET-scanning (to measure μ-receptor uptake of endorphins in the brain)—both of which are unpleasant. Endorphin antagonists such as naloxone or naltrexone that have a special affinity for the μ-receptors have also been used in reverse causality designs to show that, when endorphin uptake is blocked, the anticipated consequence (elevated pain threshold, enhanced bonding) is absent. Because of the analgesic effects of endorphins, many studies with humans have used changes in pain threshold as an effective proxy for endorphin upregulation [73,74].

**Figure 4.**
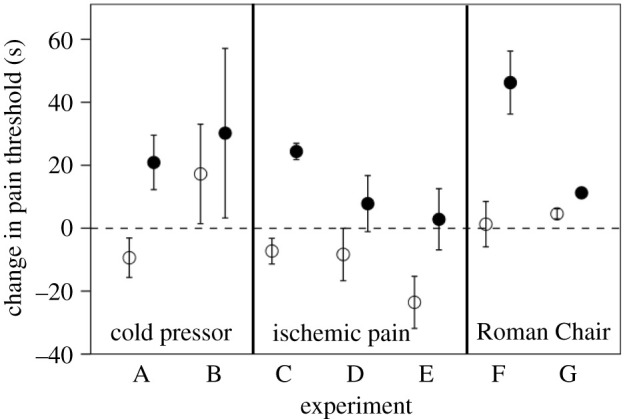
Results from seven experiments to test for endorphin upregulation following laughter. The dependent variable is the mean (±1 s.e.) change in pain threshold (after minus before) following an intervention designed to trigger laughter (filled symbols) or no laughter (control condition: unfilled symbols). The dashed line indicates that there was no change in pain threshold. Positive values indicate higher pain tolerance after the intervention (signalling endorphin uptake); negative values indicate tolerance is lower afterwards (no endorphin uptake). Pain threshold assays were determined by the duration for which a painful experience could be endured: a cold pressor task in experiments A and B, ischemic pain from a mercurial sphygmomanometer (inflated to a constant pressure) in experiments C, D and E, and the Roman Chair task in experiments F and G. In experiments A-E and G, subjects watched a comedy video (experimental condition) or a factual documentary (control condition) in a laboratory setting; in experiment F, subjects watched either live stand-up comedy (experimental condition) or live drama (control condition) at the Edinburgh Fringe Festival. Experiments A and C-G were between-subject designs; B was a within-subject design. Combined sample size is *n* = 268 subjects. For details and data, see [7,18].

Figure 4 plots the change in pain threshold in seven experiments in which human subjects watched either a comedy or a neutral video. Pain thresholds were assayed using a variety of different measures, and the stimuli varied across a wide range of comedy and documentary videos (or, in one case, live performances). Since laughter is a socially contagious behaviour [3,7], subjects in these experiments watched the video stimuli in groups of at least three individuals. Although there is considerable variance in the data, there is a consistent pattern in which (i) pain threshold change is higher in the comedy condition following laughter than in the control (no laughter) condition, and (ii) the change is consistently positive in the experimental conditions and negative or overlaps zero in the control conditions. In summary, laughter elevates pain thresholds, suggesting that endorphins have been upregulated centrally.

**Figure 5.**
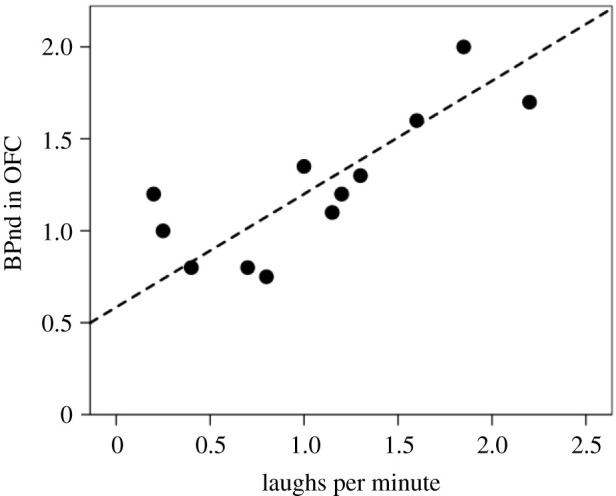
Endorphin receptor activity in the orbitofrontal cortex (OFC) (based on PET neuroimaging) as a function of the amount of laughter for individual subjects watching a comedy video between the baseline and post-viewing scans. Sample size is *n* = 12 adult males. Redrawn from [75].

To confirm that this effect is specifically because of endorphin upregulation, endorphin receptor activity was assayed before (baseline) and shortly after watching a comedy video using PET imaging [75]. Following laughter, endorphin uptake was recorded widely throughout the brain (other than the visual system), but especially so in the frontal lobes. Figure 5 shows that endorphin receptor activity in the orbitofrontal cortex is directly correlated with the frequency of laughter (*r* = 0.792, *n* = 12, *p* = 0.002). This result has since been confirmed by other neuroimaging studies [76,77]. In involuntary (or Duchenne) human laughter, the diaphragm performs a series of heavy contractions to force air from the lungs [6]. It is probably this, combined with the pain experienced when the lungs are emptied (the gasping for air after a prolonged laughter bout), that is responsible for endorphin upregulation.

**Figure 6.**
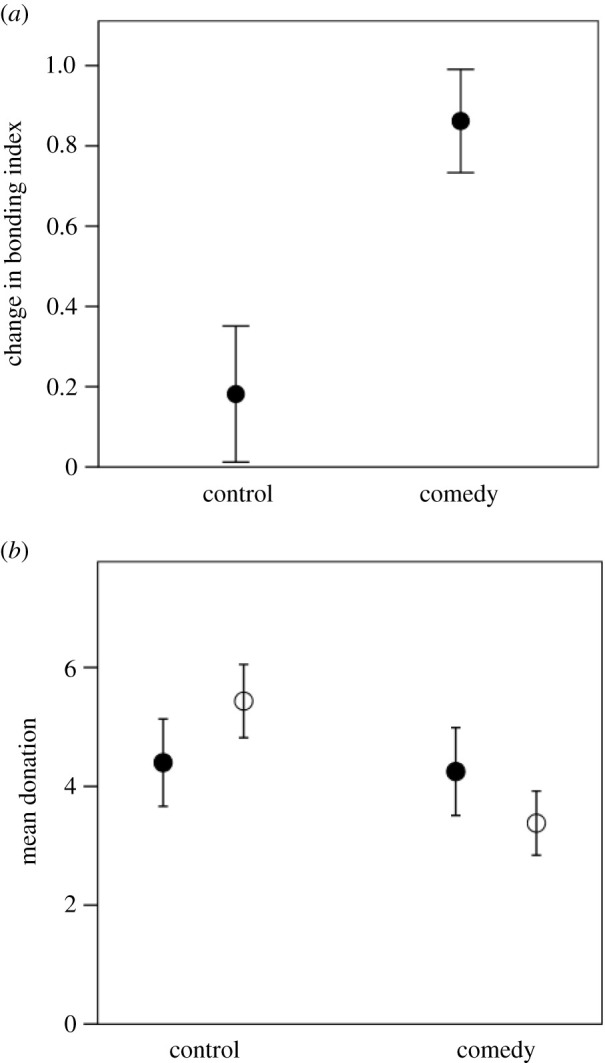
(*a*) Mean (±1 s.e.) difference in bonding index from before to after the experimental intervention in the control and comedy condition from experiment G in figure 4. The bonding index is the IOS [77]. Sample size is *n* = 50 subjects. (*b*) Monetary donations made to another group member (on a 0–10 scale) in experiments F (filled symbols) and G (unfilled symbols) in figure 3. Source [18].

The effect of laughter on bonding was determined using the standard Inclusion of Other in Self (IOS, [78]) scale with respect to other members of the experimental group (in most cases, strangers). The IOS is a simple self-rated Likert-type scale consisting of seven pairs of circles which range in equal steps from almost completely overlapping to not overlapping at all. Subjects are asked to rate their perception of their relationship with other members of the group (essentially how emotionally close they feel to them) before and after the experimental intervention. Figure 6*a* plots the change in self-rated bonding to the other subjects for experimental and control groups. Subjects in this experiment were also asked to take part in a Dictator Game with respect to one other group member at the end of the experiment: they were asked if they would like to donate part of their fee for taking part to the person on their left. Figure 6*b* plots the monetary donations made to the stranger. While the sense of bonding increases significantly after laughter, altruism does not. The two seem to belong to quite separate psychological domains.

**Figure 7.**
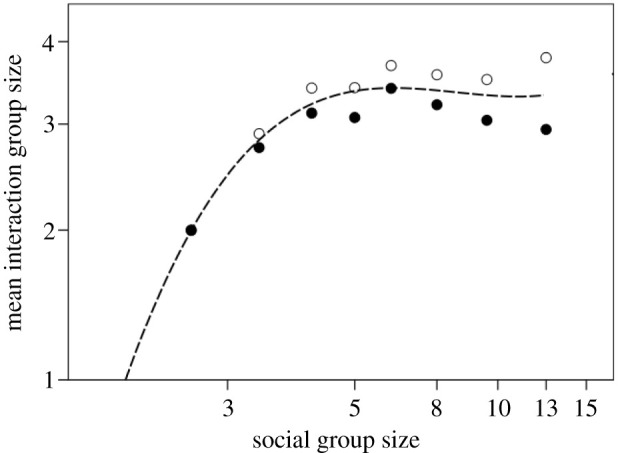
Mean size of conversation groups (unfilled symbols) and laughter groups (filled symbols) plotted against the size of the social group within which these are embedded. A laughter group is defined as all the individuals who are laughing together; conversation group size is defined as all the individuals actively engaged in a conversation with each other, either as speaker (there is only ever one) or attentive listeners; a social group includes everyone forming part of a coherent interactional group (e.g. sitting around the same table at a pub). Sample based on 450 social groups in UK, France and Germany. Source [79].

We are apt to see laughter as a large-scale phenomenon—a reflection, perhaps, of the contemporary role of comedy theatre. However, in everyday social life, laughter is a very small-scale phenomenon. Figure 7 plots the mean size of laughter and conversation groups as a function of the size of the social group within which these are embedded. A laughter group is the number of individuals who are laughing at the same time as each other and paying attention to each other, while a conversation group is the number of individuals actively involved, as speaker or listeners, in a conversation. Conversations normally have only a single speaker, and listeners display their involvement by paying attention to the speaker. The social group is all the individuals at a particular event who are sitting or standing together as a group and who are, over a period of time, involved in conversations with many or all of the individuals in that group: social groups continuously fragment into conversation subgroups of varying size [80]. Conversation group size and laughter group size are highly correlated (*r* = 0.925, *p* = 0.001). More importantly, the size of both these components rise to an asymptote at around 3.5 individuals (including the speaker). The average laughter group size is 2.7 ± 0.04 s.e. [79]. Since both speaker and audience all laugh together, this effectively means that laughter is nearly three times more efficient than grooming in terms of the number of individuals in whom endorphins can be upregulated during a given interaction.

## 4. When did human laughter evolve?

So far, we have established that, in humans, laughter upregulates central endorphins and results in an enhanced sense of bonding; but when did the peculiarly human form of laughter arise from its generic ape ancestral form? To gain some insight into likely dates, I use the classic reverse engineering approach that is commonly used in evolutionary contexts. A reverse engineering approach is a model based on first principles that seeks to identify the boundary conditions where the assumptions of the model break down (i.e. make empirically false predictions). We are interested here, in particular, in time points where there is a dramatic increase in grooming time requirement that takes grooming time beyond the limiting values of 18.5% of daytime that primates can manage. Anything beyond that will push time budgets into deficit (i.e. individuals will not be able to balance their time demands within a 12 hour day) (see [46]). These points will identify phase transitions where major changes in behaviour would have been required.

Although neither group size nor grooming time fossilize, we can estimate expected grooming time using the regression equation from figure 1 relating grooming time to group size. To do this, we first need to estimate likely group size, which we can do by interpolating through a series of equations that relate cranial volume to group size in hominoids (following [81,82]) (for justification and further details, as well as clarification on some debates around these issues, see the electronic supplementary material).

We need to be clear on three points here. First, we are not necessarily interested in the actual time a species devotes to grooming but to the time it would have needed to allocate to grooming *if this was the only way it had of bonding its group*. Second, estimates for hominin group sizes (and hence required social time) are benchmarked by the position of chimpanzees (the most social of the apes) and that of modern humans on the ape grade regression line. These, by definition, set an upper and a lower limit on the range of possibilities. All hominin fossil species must lie within this delimited range—unless we are prepared to assume that even though apes and modern humans lie on this regression line, all the hominin species in between behaved differently. A fundamental principle of all model building is to make the fewest assumptions possible and to use that as a tool for identifying the boundary conditions where the assumptions fail. This guides us to the place where we need to ask what the organism was doing that was so different to everyone else. Hence, in the absence of any compelling evidence that hominins really did behave differently to all other living hominoids (including modern humans), we must assume that the same relationship applies to them as much as to their closest living relatives. Third, and most importantly, we are less interested in the exact values than in the pattern, since it is the changes in pattern, not necessarily the absolute values, that alert us to where we should be looking. Changing the slope of the constituent equations would only change the scale on the *y*-axis, not the pattern.

**Figure 8.**
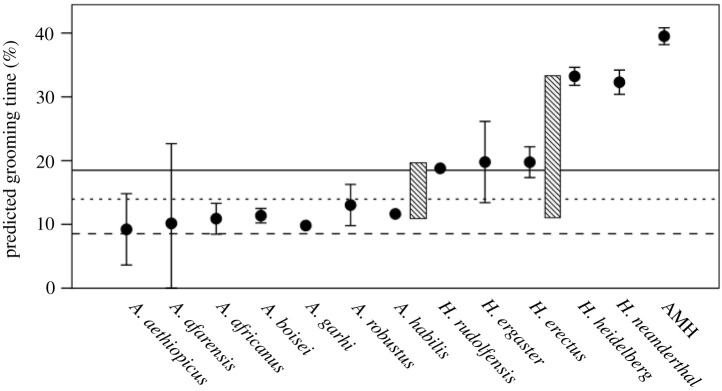
Mean (+95% confidence interval) predicted grooming time (indexed as per cent of daytime devoted to social grooming) for all fossil hominin species, estimated from individual cranial volumes (for details see the electronic supplementary material). Cranial volumes for *H. erectus* and *H. neanderthalensis* are reduced by 15% to take account of their larger visual systems [76]. AMH: fossil anatomically modern human (*Homo sapiens*). Solid line: mean predicted grooming time for early *Homo* populations; dashed line: observed mean grooming time for chimpanzees (from electronic supplementary material, table S1); dotted line: estimated social time for early *Homo* if the extra time above that for australopithecines (mean = 11%) involved laughter-based chorusing (allowing time savings of 67%). Hatched bars indicate the uplift in grooming time above the australopithecine baseline required by later hominin taxa. Note that estimates of hominin time budgets indicate that all would have been at their limit with no spare capacity [83].

The estimated grooming time values for each fossil species (including fossil *Homo sapiens*) based on individual specimens are given in figure 8; the corresponding values for cranial volume and group size from which these derive are given in the electronic supplementary material, figure S2. The values for Neanderthals (and *Homo* *erectus*) are adjusted to take account of the fact that they had much larger visual systems and hence would have had smaller neocortices for social cognition (for details, see [24,84]). The results exhibit a very clear and consistent pattern: in the australopithecines as a taxon, group size is well below the upper limit at 45 that can be bonded by social grooming, and the required grooming time itself is well below the observed upper limit at 18.5% of the day. The equations given in the electronic supplementary material would have to be very wrong for these species to have broached these limits. Thereafter, there are two major stepwise increments (indicated by the hatched bars): these coincide with the appearance of the genus *Homo* (*ca* 2.4 Ma) and the appearance of archaic humans (*ca* 600 ka). Both phase shifts represent substantial increments in required grooming time (increases of 77.3% and 195%, respectively, over the australopithecine baseline). The magnitudes of these two step changes make it unlikely that any behavioural index could offer support for both at the same time.

It is important to note that models of australopithecine time budgets (using baboon and great ape model equations to find the best biogeographic fit to the known presence/absence of australopithecines at specific locations in sub-Saharan Africa) indicate that, as with other living apes (but not most living monkeys) [58], the australopithecines were operating at maximum time budget capacity with little or no time to spare [83]. They would not have been able to increase their grooming time significantly above the observed rates in figure 8 without compromising their ability to survive (as is, in fact, also the case for chimpanzees today [85]). The subsequent species of *Homo* appear to be under even more severe time constraints [20]. This means that these later hominins would not be able to increase their social (or grooming) time allocation without first finding ways either to reduce other time budget components (i.e. feeding, moving and resting time) or to use social time more efficiently (i.e. allow more individuals to be bonded during the same time period).

If we calculate the grooming time required for the extra number of individuals (from equation (2.1)) at each of the two uplifts and divide this by the mean size of laughter groups (2.7, from figure 6), early *Homo* would have required an increment in social time of just 2.96 percentage points (equivalent to 21.3 min d^−1^) to allow group size to increase by the required 30.5 (to give an estimated group size of approximately 75), whereas archaic humans would have needed a 7.64% increase in social time (equivalent to 55 mins, or one twelfth of the day) to support their additional 77.5 group members (to give a total group size of 122). The latter would have required something much more efficient than laughter in terms of broadcast group size (i.e. the number of individuals ‘groomed’ simultaneously). Given how tightly time budgets are constrained in later *Homo* [20], spending an hour a day in sustained Duchenne laughter seems a tall order, whereas adding 20 min seems a negotiable possibility in terms of its impact on the other time budget components. An alternative possibility for later *Homo* might have been to increase the size of laughter groups so as not to use more than 2.96% extra social time. However, this would have necessitated laughter groups that were 7.64/2.96 = 2.58 times larger. To achieve this, the average size of laughter groups would have had to be 2.96 × 2.58 = 6.97 individuals, which is clearly substantially larger than is even the case in modern humans. It would seem implausible to argue that laughter group size increased but then later decreased so dramatically: evolution does not usually work like that.

In summary, this would suggest that laughter most likely emerged to solve the bonding demands of early *Homo* rather than those of archaic humans. If so, then it evolved long before language, which would accord with the fact that language is not essential for laughter [3]. The earliest likely date for the origin of speech (and hence any form of language) is the appearance of archaic humans *ca* 600 ka [19,24,86,87]. Note that the diaphragm and chest wall movements involved in Duchenne laughter are very different from those used in speech (the first involves heavy pumping; the second slow, controlled exhalations [7]). MacLarnon & Hewitt [88] suggest that the capacity to expel air forcefully from the lungs (as in coughing, but also laughter) is an adaptation for clearing the lower airways of food debris, and might have evolved as early as *Homo ergaster* (i.e. around 2.4 Ma) as part of the remodelling of the body for a new form of striding bipedal locomotion.

This timing coincides with the point at which the hominin lineage evolved a more nomadic lifestyle in open, terrestrial, high predation-risk habitats—a problem that primates generally solve by living in larger, better bonded social groups [26,56,57]. This coincided with a dramatic increase in the size of the species' biogeographic range, including, for the first time, expansion into hotter lowland habitats and the crossing of the landbridges out of Africa into Eurasia. Prior to that, australopithecines had been confined to much cooler, high altitude habitats along the East African Rift Valley fault and its extension into the high veld habitats of southern Africa [89].

## 5. Conclusion

In modern humans, laughter functions as one of a suite of behavioural mechanisms for social bonding that trigger the central endorphin system. Such behaviours do not increase the tendency to act altruistically toward others, but rather influence the sense of belonging to a group and feelings of closeness to social partners in order to maintain social group cohesion. It seems most likely that this capacity evolved early in the hominin lineage at a time associated with the appearance of the genus *Homo*, when an increase in group size was required to enable a dramatic expansion of the species’ biogeographic range into more predator-risky habitats. An early (pre-language) date for laughter would be concordant with the facts that Duchenne laughter does not require language (even though it can be stimulated linguistically using jokes), that laughter is highly contagious and that it uses a very different muscular control to that used in speech. This suggests that it may have evolved from ape laughter (an individual signal) as a form of intimate group chorusing (to which everyone contributes).

Reverse engineering models of the kind deployed here have played a seminal role in evolutionary studies, where they have allowed pinch points and constraints to be identified. While it is always possible to question the exact values predicted by any model, we are here much less interested in these than in the pattern across time. What the model tells us is that there are only two likely pinch points where a dramatic change (or phase shift) occurs in bonding demand and, of these, the more likely is the earlier. Thus, the model points to a very specific hypothesis: laughter evolved with early *Homo* and not later. To test this, we need to identify specific anatomical markers that can be detected in the fossil record in the same way that markers for speech have been used to identify when speech might have evolved [85,87,88]. For the latter, the diameter of the thoracic vertebral canal was a crucial index [88]. Since different muscles are involved in laughter (associated with rapid contraction of the lungs rather than the slow controlled exhalations characteristic of speech), these would be likely to exit at different levels in the spinal column, and hence easy to measure. Unfortunately, although the central neural mechanisms involved in laughter have been well studied, the peripheral neural mechanisms that produce laughter are very poorly understood. Whereas breath control depends on thoracic nerves T_3–5_, both laughter and coughing involve vigorous contraction of the diaphragm, supported by contraction of the stomach muscles [8]. There is some evidence, at least for coughing, that this is under the control of the phrenic (C_4_) and vagus (C_10_) cranial nerves [90], with the phrenic innervating the diaphragm musculature [91]. If these are the appropriate markers for laughter, then we might expect these to exhibit a similar pattern to the thoracic nerves in terms of enlargement in humans. While the switch in the thoracic nerve occurs uncontroversially with the appearance of archaic humans [88], figure 8 would predict that any enlargement in the cranial nerves required to support laughter should have happened 2 Myr earlier with the appearance of *H. ergaster*.

## Data Availability

Data are provided in the electronic supplementary material [92]. Otherwise, all data are sourced from indicated published papers.
